# Continuous Fascia Iliaca Compartment Block Improves Outcomes in Hip Fragility Fracture Patients

**DOI:** 10.7759/cureus.92634

**Published:** 2025-09-18

**Authors:** Matthew L Mitchell, Shahad Alassal, Gregory Panza, Edmund T Takata, Carla L Maffeo-Mitchell, Pranjali Kainkaryam, Mandeep Kumar, William Stuart, Aseel Walker, Kevin Finkel

**Affiliations:** 1 Department of Anesthesiology, Integrated Anesthesia Associates, Bone and Joint Institute, Hartford Hospital, Hartford, USA; 2 Department of Anesthesia Research and Development, Integrated Anesthesia Associates, Hartford Hospital, Hartford, USA; 3 Department of Research Administration, Hartford Hospital, Hartford, USA; 4 Department of Medicine, Bone and Joint Institute, Hartford Hospital, Hartford, USA

**Keywords:** fascia iliaca compartment block, fragility hip fracture, opioid reduction, postoperative delirium, post-op pain management

## Abstract

Objectives

The objective of this study was to evaluate the safety of continuous fascia iliaca compartment block (CFICB) and its efficacy in reducing postoperative pain, opioid use, and postoperative delirium in patients undergoing surgical repair of hip fragility fractures (HFF), compared with those who did not receive regional anesthesia (RA). Secondary outcomes included differences in hospital and post-anesthesia care unit (PACU) length of stay (LOS), discharge destination, hospital readmissions, and postoperative complications.

Methods

This was a retrospective cohort study conducted at a specialized bone and joint hospital. The study included patients aged ≥65 years who underwent surgical repair of HFF within 48 hours of injury following admission from the emergency department between July 2018 and August 2020. The intervention consisted of CFICB administered for HFF repair surgery. The main outcomes measured were postoperative pain scores, opioid consumption in morphine milliequivalents, and the incidence of postoperative delirium.

Results

A total of 209 patients who underwent surgical repair of HFF were included, with 96 patients receiving CFICB and 113 patients receiving no RA (control). Average pain scores at rest and during activity, maximum pain scores at rest, and postoperative and total opioid use were significantly lower in the CFICB group compared with controls (P < 0.05). PACU LOS was longer in the CFICB group (P < 0.05), while other secondary outcomes showed no significant differences between groups. Baseline dementia was a predictor of postoperative delirium; however, the overall incidence of postoperative delirium was similar between groups.

Conclusions

CFICB is a safe RA technique for elderly patients undergoing HFF surgical repair. This study is the first to demonstrate CFICB efficacy in reducing pain and opioid use in this specific population. Despite these benefits, CFICB did not significantly reduce the incidence of postoperative delirium. Further research is needed to optimize anesthetic regimens and improve additional outcomes in elderly HFF patients.

## Introduction

Hip fragility fractures (HFF) are common and represent a significant concern in the geriatric population. Alexiou et al. reported a growing prevalence of these fractures, particularly in the context of an aging global population, establishing them as a serious public health issue [1]. Studies by Braithwaite et al. and Veronese et al. highlighted that individuals over 65 years old are particularly vulnerable to HFF, with approximately 86% of all hip fractures occurring within this demographic, presumably due to age-associated osteoporosis [2,3]. HFF are defined as low-energy injuries, typically resulting from a fall from standing height or less, or from trauma that would not result in a fracture in a healthy individual [3]. In 2020, epidemiological data from the USA indicated that 20-30% of older adults experience moderate to severe injuries following a fall, including bruises, head trauma, and hip fractures, with an estimated 95% of hip fractures resulting from falls [3]. Overall, studies show that hip fractures contribute substantially to morbidity, mortality, and healthcare costs, with approximately 50% of patients unable to regain independent living after a hip fracture and a one-year post-fracture mortality rate exceeding 30% [4-6].

Effective pain management plays a pivotal role in facilitating early, successful, and enhanced recovery for all hip fracture patients. Regional anesthesia (RA) and peripheral nerve blockade are important components of perioperative pain management that have demonstrated efficacy in decreasing postsurgical pain and improving functional rehabilitation, two critical variables for preserving quality of life following a hip fracture [1]. In addition to impaired functional recovery, uncontrolled pain is a known risk factor for postoperative delirium, an acute alteration in mental status following surgery. Consequently, certain RA techniques have shown dual efficacy in improving functional rehabilitation and reducing postoperative delirium in selected patients [7,8].

The continuous fascia iliaca compartment block (CFICB) is a regional anesthetic technique used for perioperative pain management during hip fracture surgical repair. Multiple randomized controlled trials (RCTs) have demonstrated that CFICB is associated with decreased pain intensity, reduced opioid consumption, and shorter hospital length of stay (LOS) for hip fracture patients [9,10]. Furthermore, CFICB has been linked to a lower incidence of postoperative delirium in hip fracture patients with cognitive impairment [7,8].

However, the existing literature on CFICB in hip fracture patients typically includes a mixture of elderly patients with fragility and non-fragility hip fractures. A clear gap exists regarding exclusive studies in HFF patients. This study aims to address this gap, thereby significantly contributing to the understanding of CFICB’s role in this specific patient population.

## Materials and methods

Design and selection criteria

This retrospective cohort study was conducted at a specialized bone and joint hospital in the northeastern USA and received institutional review board (IRB) approval (approval E-HHC-2020-0107). The primary objective was to evaluate the safety and analgesic effectiveness of CFICB in elderly patients aged 65 years or older who sustained HFF and underwent surgical repair within 48 hours of injury. A comparison was made between patients receiving CFICB and a control group receiving standard multimodal analgesia (MMA) without RA. We hypothesized that CFICB would lead to lower postoperative pain scores, reduced opioid consumption, and fewer instances of delirium. The study also examined differences in surgical and block-related complications, hospital LOS, discharge destination, and rates of unplanned hospital readmissions between the two groups. Two patient cohorts were compared: Group 1, admitted from July 2018 to June 2019, and Group 2, admitted from July 2019 to August 2020. These periods were selected to evaluate the impact of a shift in institutional practice, specifically the introduction of CFICB with local anesthetic infusion for HFF patients, alongside broader adoption of an MMA approach for general pain management.

Data were extracted from existing electronic medical records (EMR; Epic, Epic Systems Corporation, Verona, WI, USA) and the Bone and Joint Institute database. No prospective data were collected specifically for this study. All data were securely stored in a password-protected REDCap database, with access restricted to the research team. Due to minimal risk to participants, the IRB waived the requirement for written informed consent.

Patients with periprosthetic femur fractures, a history of hip fracture, or hip fractures resulting from high-impact trauma (e.g., falls from a height greater than standing height and accidents) were excluded. Additionally, patients with incomplete data, those who received a single-shot FICB without a catheter, or patients whose block catheter dislodged prior to the planned removal date were excluded.

CFICB was performed either preoperatively or postoperatively immediately upon arrival to the post-anesthesia care unit (PACU) by a specialized regional anesthesiologist or an RA fellow under direct supervision. The procedure involved preparing the skin with a chlorhexidine gluconate-alcohol solution to maintain sterility and using an Arrow catheter kit (Arrow International, Inc., Reading, PA, USA). An ultrasound-guided approach was used to locate the fascia iliaca plane above the inguinal ligament. The anterior superior iliac spine was first identified in the sagittal plane using a linear ultrasound probe (Sonosite, Inc., Bothell, WA, USA; probe frequency 6-15 MHz), then the probe was moved medially to visualize the sartorius, iliopsoas, and internal oblique muscles. The target fascia iliaca plane was identified at the intersection of these fascial planes.

For catheter insertion, a 17-G Tuohy needle (B. Braun Medical Inc., Bethlehem, PA, USA) was advanced in-plane from inferior to superior, targeting the junction of the sartorius and internal oblique muscles. Correct needle placement was confirmed by the spread of injected normal saline beneath the fascia and above the iliopsoas muscle. Thirty milliliters of 0.25% ropivacaine with 1:400,000 epinephrine were administered via a low-pressure syringe, with aspiration every 5 mL to avoid vascular infiltration. The catheter was then threaded into the local anesthetic depot, and ultrasound confirmed its final position, followed by a second 30 mL bolus of ropivacaine with epinephrine. The catheter was secured with a sterile dressing, including LiquiBand (Cardinal Health, Waukegan, IL, USA) at the entry site, Mastisol (Eloquest Healthcare, Inc., Ferndale, MI, USA) applied in a small rectangular pattern around the entry point, and a labeled Tegaderm (3M; St. Paul, MN, USA) dressing covering the catheter site. Patients who received the block postoperatively in the PACU were observed for at least one hour before transfer to the general ward, where a continuous infusion of 0.2% ropivacaine at 10 mL/hr was initiated. Catheters were typically removed on postoperative day 2 unless otherwise documented.

Opioid consumption was quantified daily in morphine milligram equivalents (MME) throughout hospitalization for comparative analysis between groups. Use of non-opioid analgesics was also recorded to assess implementation of MMA, defined as the concurrent administration of two or more non-opioid medications or interventions with distinct mechanisms of action for pain relief. Pain levels were evaluated using a numeric pain scale, recording average, minimum, and maximum scores at rest and during activity on the day of surgery and daily up to postoperative day 3. These assessments included both static (rest) and dynamic (movement-evoked) pain, specifically during turning, walking, sitting, or standing from a chair, and physical therapy exercises.

Postoperative complications were systematically extracted from the EMR and categorized based on primary association with RA blocks, opioid administration, surgical procedure, or localized joint issues. The institution’s established protocol mandates consistent documentation in physician progress notes, with RA-related complications reported daily by the RA team. Block-related complications included catheter site infection, bleeding or hematoma at the insertion site, neuropathy, hemodynamic changes within 30 minutes of block placement, and local anesthetic systemic toxicity. Opioid-related complications were defined as acute confusion within 60 minutes of opioid administration and respiratory depression requiring intervention beyond supplemental oxygen via nasal cannula. Surgical complications included thromboembolic events (deep vein thrombosis, pulmonary embolism, myocardial infarction, cerebrovascular accident), hematoma requiring surgical correction, superficial or deep surgical site infections, sepsis, acute kidney injury (creatinine increase ≥0.3 mg/dL), and unplanned return to the operating room. Local joint complications included weakness, reduced quadriceps activity, numbness, and knee instability.

Delirium incidence was determined by either a positive Confusion Assessment Method score or a documented diagnosis of metabolic encephalopathy in the care provider’s progress notes throughout hospitalization [11]. Consistent with institutional policy, all patients underwent nursing assessments for delirium, which were subsequently confirmed by an internist. Outcome measures, including hospital LOS, discharge destination, and hospital readmissions, were extracted from medical charts for up to 60 days post-surgery. Readmissions were further categorized based on underlying cause, such as surgery-related complications, multifactorial issues, or new events unrelated to the initial admission.

Statistical methods

All data were assessed for normality of distribution. Statistical analyses were performed using IBM SPSS Statistics for Windows, Version 26.0 (Released 2018; IBM Corp., Armonk, NY, USA), with a P-value of less than 0.05 considered statistically significant. Continuous variables that were normally distributed were summarized as means and SDs, whereas continuous variables that were not normally distributed were summarized as medians and IQRs. Categorical data were presented as frequencies.

The study examined several outcomes. Primary outcomes included postoperative opioid use, incidence of delirium, and self-reported pain scores. Secondary outcomes included opioid-related adverse events, surgery-related adverse events, hospital LOS, discharge destination, and hospital readmissions.

Categorical variables were compared between groups using chi-square analysis; Fisher’s exact test was applied when expected cell counts were less than 5. For continuous variables, independent samples t-tests were used for normally distributed data, and Mann-Whitney U tests were used for non-normally distributed data.

Multivariate regression analysis was conducted with CFICB as the primary predictor to examine its association with primary outcomes while controlling for relevant covariates. Variables were included in the model if they were significantly different between groups in univariate analysis (P < 0.05) or demonstrated potential to explain variance in the outcome (P < 0.1). These variables included age, gender, baseline dementia, MMA use, and the Charlson Comorbidity Index (CCI). Linear regression was used for continuous dependent variables, and logistic regression was used for categorical dependent variables.

A subgroup analysis was performed, excluding patients who did not receive MMA, to evaluate whether primary outcomes differed within this cohort. Additionally, a post hoc exploratory regression analysis was conducted to examine the relationship between baseline dementia and postoperative delirium.

## Results

The study analyzed 759 patients who underwent surgical repair for HFF between July 2018 and August 2020. Of these, 385 patients received CFICB as part of a multimodal pain management strategy between July 2019 and August 2020, with 96 (24.9%) meeting the study’s inclusion criteria. Conversely, 374 HFF patients received standard MMA without CFICB between July 2018 and June 2019, of whom 113 (30.2%) were eligible for inclusion. Ultimately, a total of 209 patients who underwent surgical HFF repair were included in the study. Baseline demographics and characteristics, including age, race, ethnicity, surgical duration, CCI, and baseline diagnosis of dementia, were comparable between the CFICB and control groups, with no significant differences, as detailed in Table 1.

**Table 1:** Patient demographics and characteristics Categorical data are presented as frequency (%) and were compared using chi-square analysis or Fisher’s exact test when cell counts were less than 5. Bonferroni correction was applied for variables with multiple categories. Continuous data are presented as means (SD) and were compared using independent samples t-tests. A P-value < 0.05 was considered statistically significant. CCI, Charlson Comorbidity Index; CFICB, continuous fascia iliaca compartment block group; Control, non-FICB standard analgesia group

Variable	CFICB (n = 96)	Control (n = 113)	Total (n = 209)	P-value
Age, mean ± SD	84.52 ± 8.17	84.22 ± 8.18	84.36 ± 8.16	0.79
Gender, n (%)				0.74
Female	72 (75.0)	87 (77.0)	159 (76.1)	
Male	24 (25.0)	26 (23.0)	50 (23.9)	
Race, n (%)				0.28
White or Caucasian	83 (86.5)	103 (91.2)	186 (89.0)	
Black or African American	3 (3.1)	1 (0.9)	4 (1.9)	
Asian	2 (2.1)	0 (0.0)	2 (1.0)	
Other	7 (7.3)	9 (8.0)	16 (7.7)	
Unknown	1 (1.0)	0 (0.0)	1 (0.5)	
Ethnicity, n (%)				0.29
Hispanic or Latino	6 (6.3)	6 (5.3)	12 (5.7)	
Non-Hispanic or Non-Latino	88 (91.7)	107 (94.7)	195 (93.3)	
Unknown	2 (2.1)	0 (0.0)	2 (1.0)	
Duration of surgery, min, mean ± SD	80.34 ± 30.63	80.67 ± 30.89	80.51 ± 30.68	0.94
Baseline dementia, n (%)	40 (41.7)	34 (30.1)	74 (35.4)	0.08
CCI				
Weighted score, mean ± SD	3.56 ± 3.22	3.19 ± 3.09	3.36 ± 3.15	0.39
Weighted index, n (%)				
0	8 (8.3)	19 (16.8)	27 (12.9)	0.07
1-2	40 (41.7)	37 (32.7)	77 936.8)	0.16
3-4	21 (21.9)	29 (25.7)	50 (23.9)	0.50
≥5	27 (28.1)	28 (24.8)	55 (26.3)	0.61

The CFICB group experienced significantly lower average pain scores at rest and during activity, as well as lower maximum pain scores at rest, compared with the control group (P < 0.05). There were no significant differences between the groups in minimum pain scores at rest or during activity, nor in maximum pain scores during activity (Table 2).

**Table 2:** Self-reported numeric pain scores (0-10) ^*^ Pain scores were missing for four patients in the CFICB group. Data are presented as mean (SD) and were compared using independent samples t-tests. A P-value < 0.05 was considered statistically significant (bold values). CFICB, continuous fascia iliaca compartment block group; Control, non-FICB standard analgesia group

Variable mean ± SD	CFICB (n = 92)^*^	Control (n = 113)	Total (n = 209)	P-value
Average pain score at rest	1.77 ± 1.48	2.28 ± 1.49	2.06 ± 1.51	0.02
Average pain score with activity	3.05 ± 1.83	3.92 ± 1.78	3.53 ± 1.85	0.001
Minimum pain score at rest	0.00 ± 0.00	0.06 ± 0.41	0.03 ± 0.30	0.15
Minimum pain score with activity	0.29 ± 1.01	0.55 ± 1.14	0.44 ± 1.09	0.1
Maximum pain score at rest	5.72 ± 2.89	6.81 ± 2.82	6.32 ± 2.90	<0.001
Maximum pain score with activity	6.93 ± 2.47	7.53 ± 2.47	7.26 ± 2.48	0.09

Both postoperative and total opioid use, measured in MME, were significantly lower in the CFICB group compared with the control group (P < 0.05 and P = 0.01, respectively). Multivariate regression analysis indicated that the treatment group was a significant predictor of average resting pain score, activity pain score, and maximum pain score at rest (P < 0.05), consistent with the primary analysis of pain scores. When controlling for potential confounders, CFICB was not a significant predictor of postoperative or total MME (P > 0.05). However, using stepwise regression to remove nonsignificant predictors, CFICB became a significant predictor of postoperative MME (B = 14.269, 95% CI = 0.643, 27.895; P = 0.04), whereas total MME remained nonsignificant (B = 15.246, 95% CI = -1.084, 31.575; P = 0.07).

No significant differences were observed between the CFICB and control groups in the incidence of thrombotic events, hematoma, surgical site infection, sepsis, kidney injury, or unplanned return to the operating room. Similarly, there were no differences in local joint complications, including weakness, decreased quadriceps activity, numbness, or knee buckling that interfered with participation in physical therapy (P > 0.05). The incidence of opioid- or surgery-related complications was also similar between groups (P > 0.05). All complications that occurred were temporary, resolved prior to discharge, and none were directly attributable to the block.

Baseline dementia was a significant predictor of postoperative delirium (OR = 2.817, 95% CI = -1.084, 31.575; P < 0.001). Although the number of CFICB patients with baseline dementia was higher than in the control group (40 (42%) vs 34 (30%), respectively), the frequency of postoperative delirium was similar between groups (Table 3).

**Table 3:** Postoperative complications, including opioid-related, surgery-related, and local joint complications ^*^ The breakdown of events equals the “total” because this represents the total number of patients with at least one event. Data are presented as frequency (%) and were compared using chi-square analysis or Fisher’s exact test when cell counts were less than 5. A P-value < 0.05 was considered statistically significant. CFICB, continuous fascia iliaca compartment block group; Control, non-FICB standard analgesia group

Variable	CFICB (n = 96)	Control (n = 113)	Total (n = 209)	P-value
Opioid-related complications, n (%)				
Delirium or metabolic encephalopathy	33 (33.4)	34 (30.1)	67 (32.1)	0.51
Respiratory depression	1 (1.0)	0 (0.0)	1 (0.5)	0.46
Total number of patients with any opioid events^*^	33 (34.4)	33 (29.2)	66 (31.6)	0.42
Surgery complications, n (%)				
Thromboembolic event	3 (3.1)	4 (3.5)	7 (3.3)	1
Hematoma	1 (1.0)	0 (0.0)	1 (0.5)	0.46
Site infection	2 (2.0)	0 (0.0)	2 (1.0)	0.21
Sepsis	1 (1.0)	2 (1.8)	3 (1.4)	1
Kidney injury	20 (20.8)	13 (11.5)	33 (15.8)	0.07
Unplanned operating room return	2 (2.1)	1 (0.9)	3 (1.4)	0.6
Total number of patients with any surgery event^*^	27 (28.1)	19 (16.8)	46 (22.0)	0.05
Local joint complications, n (%)				
Weakness	6 (6.3)	7 (5.7)	13 (6.2)	1
Decreased quadriceps activity	1 (1.0)	1 (0.9)	2 (1.0)	1
Numbness	2 (1.0)	1 (0.9)	3 (1.4)	0.6
Knee buckling	2 (2.1)	7 (5.7)	9 (4.3)	0.18

Apart from PACU LOS, there were no significant differences between the two groups in hospital LOS, discharge destination, or hospital readmissions (P > 0.05) (Table 4).

**Table 4:** LOS, discharge destinations, hospital readmissions, and days to readmission Continuous data are presented as median (IQR) and were compared using the Mann-Whitney U test. Categorical data are presented as frequency (%) and were compared using chi-square or Fisher’s exact test when cell counts were less than 5. Bonferroni correction was applied when variables had multiple categories. A P-value < 0.05 was considered statistically significant (bold values). CFICB, continuous fascia iliaca compartment block group; Control, non-FICB standard analgesia group; LOS, length of stay; PACU, post-anesthesia care unit

Variable	CFICB (n = 96)	Control (n = 113)	Total (n = 209)	P-value
PACU LOS, minutes, median, IQR	119.00, 71.00	89.00, 53.00	97.50, 59.00	<0.001
Hospital LOS, hours, median, IQR	111.95, 42.67	106.20, 38.60	107.90, 41.90	0.24
Discharge destination, n (%)				0.59
Skilled nursing facility	87 (90.6)	107 (94.7)	194 (92.8)	
Home with health care	6 (6.3)	5 (4.4)	11 (5.3)	
Inpatient rehab facility	1 (1.0)	0 (0.0)	1 (0.5)	
VA facility	1 (1.0)	0 (0.0)	1 (0.5)	
Expired	1 (1.0)	1 (0.9)	2 (1.0)	
Total hospital readmissions, n (%)	18 (18.8)	22 (19.5)	40 (19.1)	0.80
Hospital readmission reason, n (%)	n = 18	n = 22	n = 40	0.94
Surgery	3 (16.7)	3 (13.6)	6 (15.0)	
Multifactorial	10 (55.6)	12 (54.5)	22 (55.0)	
Unrelated	5 (27.8)	7 (31.8)	12 (30.0)	
Days to readmission, n (%)	n = 18	n = 22	n = 40	0.64
30 days	11 (61.1)	15 (50.0)	26 (45.2)	
60 days	7 (38.9)	7 (31.8)	14 (23.8)	

During the 2018-2019 period, prior to the standardization of CFICB treatment, the institute was transitioning to an MMA approach. This created a disparity in treatment protocols between study groups: all patients in the CFICB group received MMA, whereas only 68% of the control group did, with the remaining 32% relying primarily on opioids for pain management. To mitigate the confounding effect of this treatment variation on primary outcomes, a subgroup analysis was conducted, excluding 36 control patients who did not receive MMA.

This subgroup analysis confirmed that significant differences persisted between the groups for postoperative and total MME, average pain scores at rest and during activity, and maximum pain score at rest, consistent with the overall study findings. Notably, the maximum pain score during activity, which was not significantly different in the unadjusted total sample, became significantly different between groups in the subgroup analysis. Furthermore, after adjusting for covariates in the subgroup analysis, maximum pain score (presumably with activity) also demonstrated a significant inter-group difference (Table 5, Table 6).

**Table 5:** Opioid consumption measured by MME ^a ^Number after excluding control patients who did not receive MMA. ^b^ Nonparametric Mann-Whitney U test was used due to the non-normality of data. ^c^ Linear regression analysis with CFICB as the primary predictor, adjusting for age, gender, baseline dementia, and weighted CCI score. Data are presented as median (IQR). A P-value < 0.05 was considered statistically significant (bold values). CCI, Charlson Comorbidity Index; CFICB, continuous fascia iliaca compartment block group; Control, non-FICB standard analgesia group; MME, morphine milligram equivalents

Variable, median, IQR	CFICB (n = 96)	Control (n = 77)^a^	Total (n = 173)	U	P^b^	P^c^
Postoperative MME	14.50, 40.38	37.5, 68.25	25.10, 47.50	2542	<0.001	0.006
Total MME	29.70, 53.10	56.20, 71.38	44.50, 59.95	2524.5	<0.001	0.014

**Table 6:** Adjusted self-reported numeric pain scores (0-10) ^a^ Pain scores were missing for four patients in the CFICB group. ^b^ Number after excluding control patients who did not receive MMA. ^c^ Nonparametric Mann-Whitney U test was applied after excluding patients without MMA. ^d^ Linear regression analysis with CFICB as the primary predictor, adjusting for age, gender, baseline dementia, and weighted CCI score. Data are presented as means (SD). A P-value < 0.05 was considered statistically significant (bold values). CCI, Charlson Comorbidity Index; CFICB, continuous fascia iliaca compartment block group; Control, non-FICB standard analgesia group

Variable, mean ± SD	CFICB (n = 92)^a^	Control (n = 77)^b^	Total (n = 169)	P^c^	P^d^
Average pain score at rest	1.77 ± 1.48	2.58 ± 1.58	2.14 ± 1.57	0.001	0.003
Average pain score with activity	3.05 ± 1.83	4.32 ± 1.82	3.63 ± 1.93	<0.001	<0.001
Maximum pain score at rest	5.72 ± 2.89	7.26 ± 2.77	6.42 ± 2.93	0.001	0.002
Maximum pain score with activity	6.93 ± 2.47	8.03 ± 2.38	7.43 ± 2.48	0.004	0.009
Minimum pain score at rest	0.00 ± 0.00	0.09 ± 0.49	0.04 ± 0.33	0.078	0.133
Minimum pain score with activity	0.29 ± 1.01	0.61 ± 1.20	0.44 ± 1.11	0.068	0.116

Similar to the overall sample, the subgroup analysis did not identify significant differences in the incidence of delirium or metabolic encephalopathy between the CFICB group (33.4%) and the control group (22.1%; unadjusted P = 0.09, adjusted P = 0.336).

## Discussion

HFF are relatively common in elderly patients with multiple comorbidities [12,13]. The increasing number of surgical repairs for these injuries, combined with relatively poor in-hospital and one-year postoperative mortality rates, has driven efforts to improve patient care and outcomes.

The results of this retrospective study are consistent with existing research on the use of CFICB for pain management in hip fracture patients. Compared with the control group, patients receiving CFICB reported significantly lower average and maximum pain scores, both at rest and with activity, as well as a marked reduction in opioid consumption. These findings align with those from multiple RCTs [9]. However, the absolute difference in mean average pain scores between the CFICB and control groups was relatively small, raising questions about its clinical significance. While other RCTs have shown larger effect sizes for CFICB, the overall impact remains modest [9].

Although postoperative and total opioid MME were significantly reduced in CFICB patients, this did not translate into a reduced incidence of postoperative delirium. Most of the current literature evaluating the effect of FICB on postoperative delirium likewise reports no statistically significant benefit. A prior study hypothesized that continuous FICB might reduce delirium [12], but our study did not support this. Instead, we found that presurgical dementia status was the strongest predictor of postoperative delirium, underscoring its multifactorial etiology and the need for further investigation.

Similarly, most studies have not demonstrated significant improvements in hospital LOS following FICB for hip fracture surgery [10]. In line with these findings, our study also found no statistical differences in hospital LOS, discharge destination, or hospital readmissions between the CFICB and control groups. These results highlight the need for future studies to evaluate the potential long-term benefits of CFICB.

This study further suggests that CFICB does not increase the risk of complications in patients undergoing HFF repair. Although local joint complications could theoretically be attributable to the block, their nearly equal distribution between groups suggests they are more likely related to the surgery itself. Moreover, none of these complications occurred immediately after the block procedure, further supporting the safety of CFICB as an intervention.

One limitation is that the standardization of MMA coincided with the introduction of CFICB as a standard pain management strategy for HFF. Since MMA is known to reduce opioid consumption and pain scores [2], it may have acted as a confounding factor. However, a subgroup analysis excluding non-FICB patients who did not receive MMA yielded similar results, suggesting that this did not account for the observed benefits in the CFICB group. Other limitations include the retrospective design, the single-center setting, and the comparison of cohorts treated during different time periods. These factors may introduce confounders that could have influenced the outcomes.

Importantly, this is the first study to specifically examine the effectiveness of CFICB for postoperative pain management in HFF patients, a population not previously studied for this intervention. To confirm and quantify the differences observed, future RCTs are needed. Such trials should directly compare CFICB and other RA techniques in reducing pain and opioid use in elderly patients with HFF versus those with other types of hip fractures.

## Conclusions

This study demonstrates encouraging results for the use of CFICB in patients with HFF but also highlights the need for further research to optimize pain management and reduce the risk of postoperative delirium in this vulnerable population. The findings suggest that CFICB can be a valuable component of MMA; however, additional studies are essential to develop strategies that improve outcomes such as discharge destination, return to baseline functionality, and one-year postoperative mortality.
